# Roles of lactate and lactylation in tumor immune suppression and drug resistance

**DOI:** 10.3389/fimmu.2026.1836534

**Published:** 2026-05-29

**Authors:** Jiaqi Li, Wangji Yang, Tian Xin, Zhaokai Zhou, Xiaoyu Liu, Pei Tang, Junsha An, Bin Ma, Fu Peng

**Affiliations:** 1 West China School of Medicine, West China School of Pharmacy, Department of Thyriod and Breast Surgery, West China School of Public Health, West China Fourth Hospital, Sichuan University, Chengdu, China; 2 National Clinical Research Center for Metabolic Diseases, Department of Urology, The Second Xiangya Hospital of Central South University, Changsha, Hunan, China; 3 Department of Pharmacology, Key Laboratory of Drug-Targeting and Drug Delivery System of the Education Ministry, Sichuan Engineering Laboratory for Plant-Sourced Drug and Sichuan Research Center for Drug Precision Industrial Technology, Chengdu, China

**Keywords:** drug resistance, immune suppression, lactate, lactylation, tumor microenvironment

## Abstract

Metabolic reprogramming is a hallmark of cancer, with excessive lactate accumulation driven by aerobic glycolysis profoundly reshaping the tumor microenvironment (TME). Beyond being a metabolic by-product, lactate acts as a signaling metabolite and epigenetic regulator that promotes immune suppression and therapeutic resistance. Lactate-induced acidification impairs immune cell function and reprograms macrophages, T cells, and dendritic cells toward immunosuppressive phenotypes. Moreover, lactate drives lysine lactylation of histone and non-histone proteins, linking metabolic status to transcriptional regulation and oncogenic signaling. Emerging evidence indicates that lactate and lactylation cooperatively enhance tumor survival, invasion, and resistance to chemotherapy, radiotherapy, anti-angiogenic therapy, and immunotherapy. This review summarizes mechanisms of lactate production, transport, and signaling, and discusses therapeutic strategies targeting lactate metabolism, lactylation, and TME acidification, highlighting their potential in precision and combination cancer therapy.

## 1 Introduction

Metabolic dysregulation in tumor cells is a key feature of cancer biology. During rapid proliferation, tumor cells undergo metabolic reprogramming, with the most notable characteristic being the Warburg effect (1, 2). This effect refers to the phenomenon where tumor cells rely on aerobic glycolysis to generate energy, even in the presence of ample oxygen, rather than utilizing the more efficient oxidative phosphorylation process. Despite the lower efficiency of this metabolic process, it provides tumor cells with the necessary precursors to rapidly synthesize biomolecules such as fatty acids and nucleotides, thereby supporting their rapid proliferation (3).

Lactate is the primary by-product of glycolysis. Under normal circumstances, pyruvate enters the mitochondria for oxidation, but in tumor cells, pyruvate is converted to lactate. Lactate not only acts as a by-product of energy metabolism, but also plays a crucial role in tumor microenvironment (TME). By promoting acidification of TME, lactate further enhances the invasiveness and metastatic potential of tumor cells (4). Accumulation of lactate leads to local acidosis, which suppresses the function of immune cells and boosts the immune evasion capabilities of tumor cells. Lactate also regulates various signaling pathways within TME, influencing tumor growth, angiogenesis and resistance to therapies (5). Additionally, lactate itself can influence cellular function and tumor progression through the modulation of protein lactylation.

Lactylation is a newly discovered epigenetic modification in which lactate molecules attach to lysine residues of proteins, forming a post-translational modification. Lactylation is not only involved in regulating tumor cell metabolism but is also closely linked to gene expression, immune response and cell signaling processes (6).

As a result, the study of the role of lactate and lactylation in tumors has become a research hotspot in cancer biology in recent years (7). Lactylation modification provides new molecular targets for tumor development and offers potential intervention pathways for targeted therapy. Lactate, as a metabolic product, and its effect through lactylation modifications may become a novel target for cancer treatment. Lactate’s influence on tumor immune evasion also encourages a re-evaluation of immunotherapy strategies, providing new perspectives and breakthroughs for future cancer treatments.

In summary, lactate not only accumulates as a metabolic byproduct but also acts as a key signaling metabolite that induces lactylation, thereby translating metabolic states into epigenetic programs that ultimately promote immunosuppression and therapeutic resistance.

## 2 Lactate homeostasis in tumor cells

### 2.1 Metabolism of lactate

In tumor cells, the source of lactate is closely related to the cell’s metabolic pathways and is primarily produced through aerobic glycolysis and glutamine catabolism. Lactate accumulation is one of the typical features of tumor metabolism and this phenomenon is closely associated with characteristics such as tumor proliferation, invasion and immune evasion.

Firstly, aerobic glycolysis is one of the main pathways through which tumor cells produce lactate. During glycolysis, glucose is broken down into pyruvate, and pyruvate is converted into lactate by lactate dehydrogenase (LDH). Tumor cells generate lactate through this pathway to maintain rapid energy production and metabolic demand even under hypoxic conditions (8).

The high levels of lactate in tumor cells are related to the balance between aerobic glycolysis and pyruvate dehydrogenase (PDH) flux. PDH is a key enzyme involved in the oxidative decarboxylation of pyruvate to acetyl-CoA, which then enters the tricarboxylic acid (TCA) cycle. In normal cells, PDH flux is high and pyruvate enters the mitochondria for oxidative metabolism. In many tumor cells, however, the activity of PDH is inhibited, preventing pyruvate from entering the TCA cycle and causing it to be converted into lactate through LDH (9). Thus, although tumor cells are in an oxygen-rich environment, they still choose to generate lactate via glycolysis, maintaining their high rate of proliferation.

In addition, glutamine is another important source of lactate in tumor cells. Tumor cells often use glutamine catabolism to provide amino acids and energy. During glutamine metabolism, glutamine is converted into α-ketoglutarate, which enters the TCA cycle, supplying energy to tumor cells. However, glutamine metabolism can also produce lactate, especially under hypoxic or high demand conditions, where glutamine metabolism further promotes lactate accumulation (10, 11).

Tumor cells produce lactate through pathways such as aerobic glycolysis, regulation of PDH flux and glutamine catabolism. Lactate is not only a by-product of tumor cell energy metabolism, but also plays a critical role in TME, supporting tumor growth and metastasis.

### 2.2 The lactate shuttle and the signaling pathways

**Figure 1:**
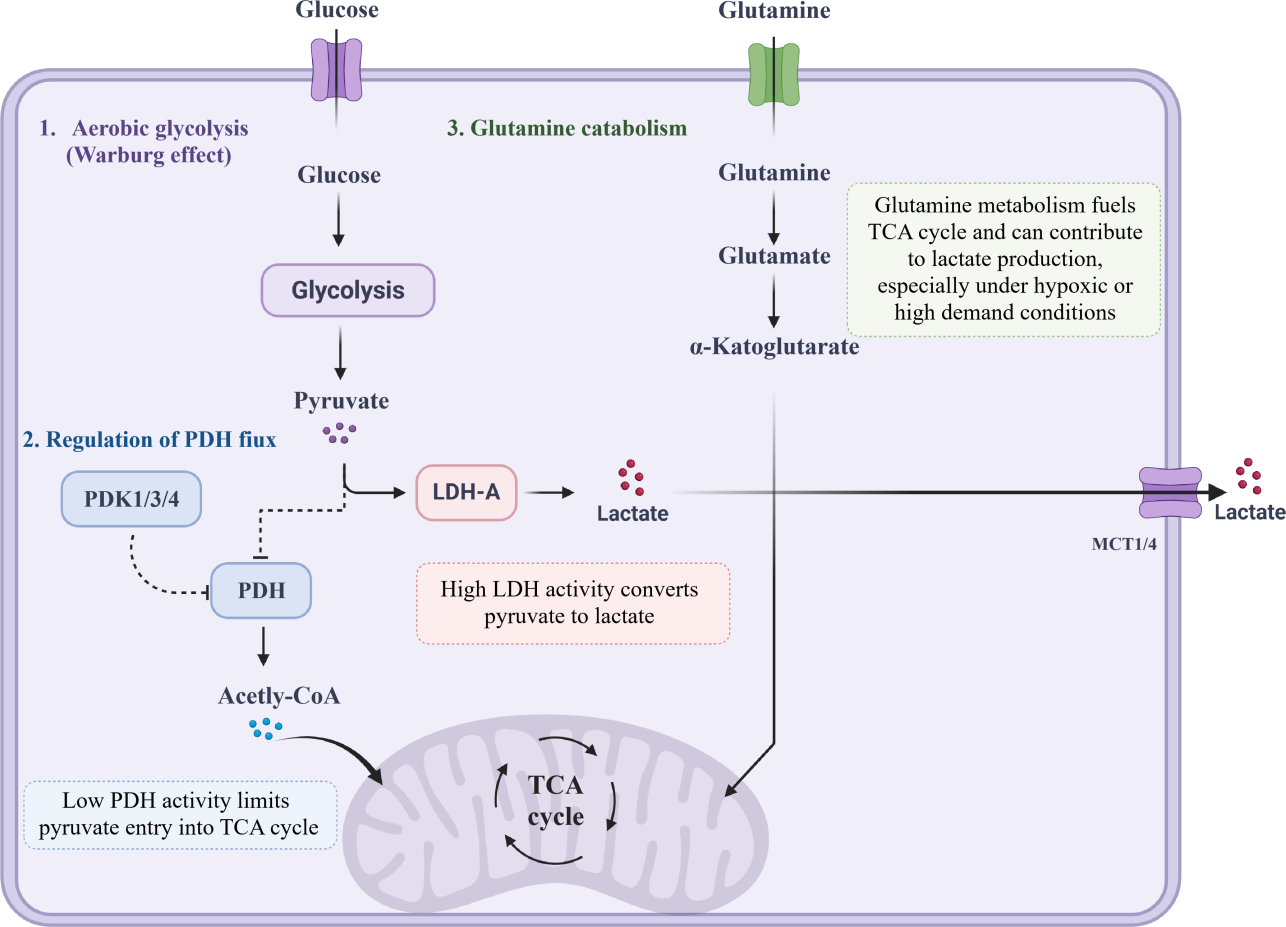
Glycolytic pathway and lactate production in tumor cells.

Once produced, lactate is transported across the cell membrane by monocarboxylate transporters (MCTs), including subtypes such as MCT1, MCT2 and MCT4. Among these, MCT1 and MCT4 are particularly important in tumor cells (12). MCT1 primarily facilitates the influx of lactate, while MCT4 exports accumulated lactate from the cell, as shown in **Figure 1** (13). The transport of lactate helps to maintain the acid-base balance within and outside tumor cells, thus sustaining the acidic environment in TME. Tumor cells export lactate via MCT4 to prevent its accumulation within the cell, thus avoiding disruption of cellular function due to the acidic environment, while the accumulation of lactate also contributes to the acidification of TME.

LDH is the key enzyme in lactate production, particularly the LDH-A isoform, which plays a critical role in glycolysis (14). LDH catalyzes the conversion of pyruvate to lactate, maintaining the metabolic state of tumor cells. Tumor cells often increase lactate production by upregulating LDH-A expression. This process not only supports rapid tumor cell proliferation but also regulates TME through lactate accumulation (15). Regulation of LDH expression in tumors is significant because it facilitates the adaptive metabolic reprogramming of tumor cells, allowing them to survive and proliferate in hypoxic and acidic environments (16).

Lactate metabolism in tumor cells is regulated by various transcription factors, especially HIF-1α and c-Myc. HIF-1α is activated under hypoxic conditions, promoting the up-regulation of glycolytic pathways and increasing lactate production (17, 18). At the same time, HIF-1α also regulates the expression of MCT4, which promotes the export of lactate. On the other hand, c-Myc enhances glucose uptake and lactate generation by promoting metabolic reprogramming, further driving the rapid growth of tumor cells (19).

In addition to glycolysis, tumor cells also rely on other metabolic pathways, such as fatty acid synthesis and amino acid metabolism, which intersect with lactate metabolism, forming a complex metabolic network. Tumor cells provide the necessary carbon sources and energy through fatty acid synthesis and amino acid metabolism, which are closely related to the generation and clearance of lactate, collectively supporting the proliferation and survival of tumor cells (20).

Lactate can also act as a metabolic intermediate involved in various signaling pathways. By binding to specific receptors, lactate regulates the physiological and pathological states of cells. Among the most common lactate signaling pathways are those activated by the lactate receptor (such as GPR81, also known as HCA1) (21). Upon binding to GPR81, lactate activates G protein-coupled receptor (GPCR) signaling pathways, triggering a decrease in cAMP levels and further regulating intracellular metabolic activities by inhibiting adenylate cyclase (22). This pathway plays an important role in the regulation of glucose metabolism, anti-inflammatory responses and cellular acid-base balance. Additionally, lactate can also increase the stability of HIF-1α and initiate hypoxia response pathways that help cells adapt to low-oxygen environments and regulate biological processes such as angiogenesis and glycolysis. Lactate therefore not only serves as a metabolic by-product, but also functions as an important signaling molecule involved in various physiological processes.

## 3 Lactylation in tumor

### 3.1 Occurrence of lactylation

Lactylation is a post-translational protein modification process that involves the accumulation of lactate to modify lysine residues on histones during cellular metabolism. Lactylation is a global modification in human cells and is classified into histone lysine lactylation and non-histone lysine lactylation (23).

In mammalian systems, lactoyl-CoA has been identified as a key metabolic intermediate supporting lysine lactylation. Although present at relatively low abundance compared with major acyl-CoAs, lactoyl-CoA exists at levels comparable to other regulatory acyl-CoAs, supporting its functional relevance in epigenetic regulation.

Lysine lactylation (Kla) has also been identified in various eukaryotic cells and is particularly enriched in cancer. Bioinformatics analysis showed that these lactylated proteins were evolutionarily conserved and involved in various cellular functions like energy metabolism, gene regulation, and protein biosynthesis. Notably, lactylation levels are significantly elevated in tumor tissues compared with adjacent normal tissues and are closely associated with aggressive tumor phenotypes and poor clinical outcomes.

### 3.2 Regulatory mechanisms of lactylation

**Figure 2:**
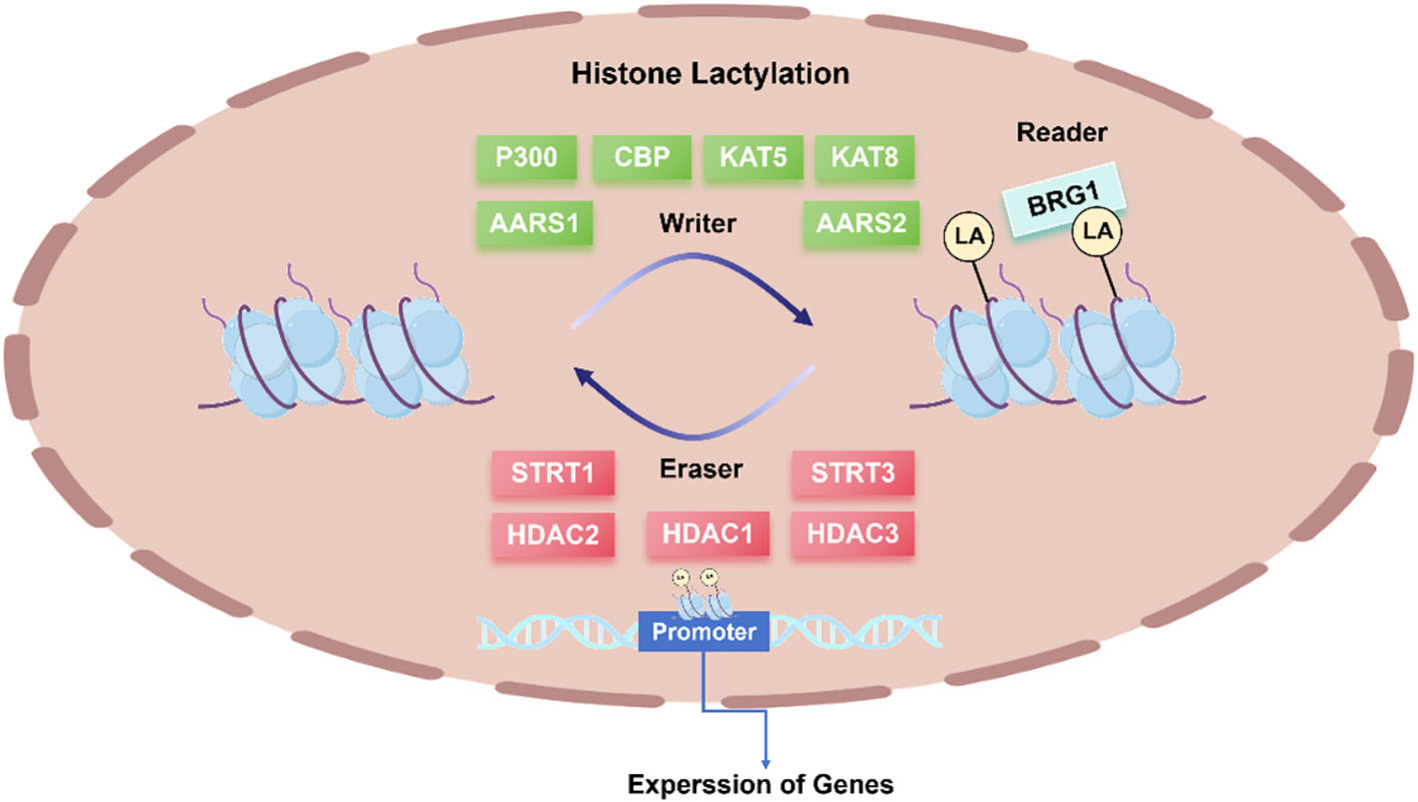
Schematic diagram of the “writer-reader-eraser” regulatory mechanism of histone lactylation. Lactate (LA) serves as the substrate. Writer enzymes (including P300, CBP, KAT5, KAT8, AARS1, and AARS2) catalyze the formation of histone lactylation; eraser enzymes (including SIRT1, SIRT3, HDAC1, HDAC2, and HDAC3) remove this modification; reader proteins (e.g., BRG1) recognize lactylated sites and subsequently regulate gene expression at downstream promoter regions.

Lactylation is regulated by specific lysyltransferases, known as ‘writers’, and delactylases, referred to as ‘erasers’, and further specifically recognized and bound by effector proteins called ‘readers’, as summarized in **Table 1** and **Figure 2** (24).

**Table 1:** Writers, erasers, and readers involved in protein lactylation.

Category	Protein class	Representative proteins	Functional role in lactylation
Writer	Histone acetyltransferases (HATs)	p300, CBP, KAT5, KAT8	Catalyze the transfer of lactyl groups to lysine residues, linking cellular lactate levels to epigenetic regulation
	Aminoacyl-tRNA synthetases	AARS1, AARS2	Non-canonical writers implicated in protein lactylation, potentially coupling metabolic state with translational regulation
Eraser	Classical histone deacetylases	HDAC1, HDAC2, HDAC3	Remove lysine lactylation marks and maintain the dynamic reversibility of this modification
	NAD^+^ -dependent deacetylases (Sirtuins)	SIRT1, SIRT3	Delactylation under metabolic and stress conditions, linking lactylation to energy sensing pathways
Reader	PTM-recognition and chromatin-remodeling proteins	BRG1	Recognize lactylated lysine residues and mediate downstream chromatin remodeling and transcriptional regulation

Writers (lactyltransferases) are responsible for catalyzing the transfer of lactyl groups to lysine residues on histone and non-histone proteins. Among them, histone acetyltransferases (KATs) are currently recognized as the major enzymatic drivers of lactylation. The transcriptional coactivator p300/CBP was the first identified lactylation “writer”, capable of utilizing lactyl-CoA as a donor to catalyze histone lysine lactylation, such as H3K18la and H4K12la (25, 26). Functional studies demonstrated that p300 depletion significantly reduces global lactylation levels, whereas its overexpression enhances lactylation, supporting its central role in this modification (27).

Beyond p300/CBP, additional enzymes have been identified as potential lactyltransferases, including members of the MYST family (e.g., KAT5/TIP60, KAT7/HBO1, and KAT8), GNAT family proteins (e.g., GCN5/KAT2A), and noncanonical enzymes such as alanyl-tRNA synthetase (AARS1/2) (25). These enzymes exhibit substrate specificity toward both histone and non-histone proteins, indicating that lactylation is not limited to chromatin regulation but also participates in diverse cellular processes such as metabolism, DNA repair, and immune signaling (28).

Erasers (delactylases) remove lactyl modifications and restore the unmodified lysine state, thereby ensuring the reversibility of lactylation. Current evidence suggests that classical histone deacetylases (HDAC1–3) and NAD^+^-dependent sirtuins (SIRT1–3) function as major lactylation “erasers” (26). For example, HDAC family members have been shown to remove histone lactylation marks such as H3K9la, while SIRT2 and SIRT3 regulate lactylation of both nuclear and mitochondrial proteins, linking lactylation dynamics to cellular metabolic states (28). However, compared with acetylation, the enzymatic specificity and substrate selectivity of lactylation erasers remain incompletely characterized.

Readers are proteins that recognize lactylated lysine residues and translate this modification into downstream biological effects, such as transcriptional activation or repression. Emerging studies have identified several candidate lactylation “readers”, including DPF2, TRIM33, and BRG1, which bind to lactylated histones and regulate gene expression programs associated with tumor progression and immune suppression. Nevertheless, the identification of lactylation readers is still in its early stages, and the structural basis for lactylation recognition remains largely undefined.

Importantly, compared with other well-characterized epigenetic modifications such as acetylation, the regulatory network of lactylation is far less understood. In particular, the limited knowledge of reader proteins and the potential redundancy among writers and erasers highlight a critical gap in the field. Future studies are required to systematically identify lactylation-specific regulatory proteins and to clarify their context-dependent functions in cancer and immune regulation.

### 3.3 Crosstalk between lactylation and other regulatory layers

Emerging evidence indicates that lactylation does not function in isolation but instead participates in multilayered crosstalk with other regulatory mechanisms, including classical epigenetic modifications, RNA modifications, and intracellular signaling pathways.

At the epigenetic level, lactylation exhibits extensive interplay with lysine acetylation. Both modifications are derived from glycolytic intermediates—lactate and acetyl-CoA—and frequently occur on overlapping lysine residues of histone and non-histone proteins. Previous proteomic analyses have shown that a substantial proportion of lactylated proteins are also acetylated, suggesting potential competition or coordination between these modifications at shared sites (29). This metabolic dependency implies that fluctuations in intracellular metabolite availability can dynamically shift the balance between acetylation and lactylation, thereby fine-tuning chromatin accessibility and transcriptional outputs. Importantly, the interaction between lactylation and acetylation can be either competitive or cooperative. In macrophages, histone acetylation is rapidly induced during early inflammatory activation, whereas histone lactylation accumulates at later stages, promoting the expression of reparative genes such as Arg1. This temporal switch highlights a coordinated epigenetic transition from pro-inflammatory to immunoregulatory states.

Beyond classical epigenetic crosstalk, lactylation also interfaces with RNA modification pathways, particularly N6-methyladenosine (m6A) regulation. In ocular melanoma, histone lactylation has been shown to promote tumorigenesis by upregulating the expression of the m6A reader protein YTHDF2. YTHDF2 subsequently recognizes m6A-modified transcripts such as PER1 and TP53 mRNAs and facilitates their degradation, thereby accelerating tumor progression. This finding reveals a previously unappreciated axis linking metabolic reprogramming, histone lactylation, and post-transcriptional RNA regulation (30).

In addition, lactylation is closely integrated with oncogenic signaling pathways, forming feedback regulatory circuits. In clear cell renal cell carcinoma (ccRCC), loss of von Hippel–Lindau (VHL) function correlates with increased histone lactylation. Mechanistically, VHL inactivation induces histone lactylation–dependent transcriptional activation of platelet-derived growth factor receptor β (PDGFRβ), while PDGFRβ signaling in turn further enhances lactylation levels, forming a positive feedback loop that drives tumor progression (31).

These findings collectively suggest that lactylation functions as a central regulatory node that bridges metabolism, epigenetic remodeling, RNA processing, and signaling transduction. However, despite these advances, the molecular principles governing such multilayered crosstalk remain incompletely understood. In particular, how cells prioritize among competing acylation modifications and integrate signals across epigenetic and post-transcriptional layers under different metabolic conditions remains to be elucidated.

## 4 Biological functions of lactate and lactylation in tumors

**Figure 3:**
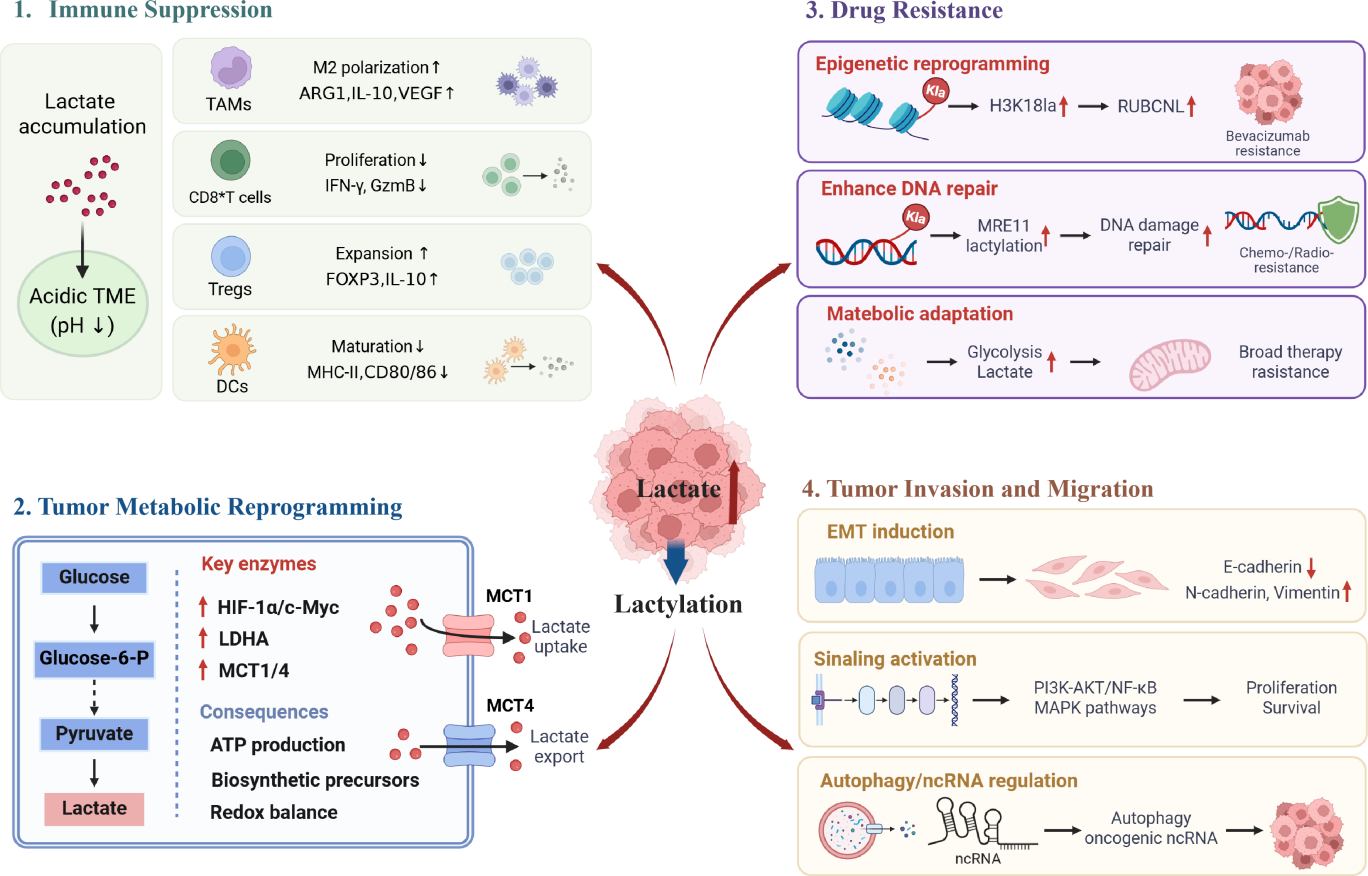
Role of lactylation in tumors. Lactic acid exerts four major oncogenic roles in the tumor microenvironment. (1) Immune suppression: lactate accumulation and the consequent acidic pH promote M2 polarization of tumor-associated macrophages (upregulating ARG1, IL-10, VEGF) while reducing their proliferation (downregulating IFN-γ, GzmB); suppress CD8^+^ T cell function; expand regulatory T cells (upregulating FOXP3, IL-10); and impair dendritic cell maturation (downregulating MHC-II, CD80/86). (2) Metabolic reprogramming: driven by HIF-1α/c-Myc, key enzymes such as LDHA and MCT1/4 convert glucose-derived pyruvate into lactate, thereby supporting ATP production, biosynthetic precursors, and redox balance. (3) Drug resistance: lactate-derived H3K18la upregulates RUBCNL leading to bevacizumab resistance; lactylation of MRE11 enhances DNA damage repair causing chemo-/radio-resistance; and increased glycolysis with lactate production further sustains metabolic adaptation for resistance. (4) Tumor invasion and migration: lactic acid activates PI3K-AKT, NF-κB, and MAPK pathways to promote proliferation and survival; MCT1-mediated lactate uptake drives lactylation and regulates autophagy/oncogenic non-coding RNAs, while also inducing epithelial–mesenchymal transition (EMT).

Lactate and lactylation have emerged as key regulators in tumor biology, exerting profound and multifaceted effects on cancer progression. By remodeling the tumor immune microenvironment, promoting therapeutic resistance, enhancing tumor invasion and metastasis, and facilitating immune evasion, they play pivotal roles in shaping tumor development and clinical outcomes, as shown in **Figure 3**.

### 4.1 Regulation of tumor metabolism

Lactylation has been shown to have a significant impact on tumor metabolism. In colorectal cancer, ULK2, a serine/threonine protein kinase, has been found to promote migration and invasion of cancer cells via MCT4-mediated lactate export. ULK2 overexpression was associated with EMT-like phenotypic alterations, including loss of membranous E-cadherin and β-catenin nuclear accumulation, which collectively promoted CRC invasion. Furthermore, ULK2 upregulated MCT4 expression on the plasma membrane, resulting in increased extracellular lactate levels and enhanced invasive capacity *in vitro (*32).

In hepatocellular carcinoma (HCC), Glypican-3 (GPC3) knockdown decreased the cell viability, stemness, glucose uptake, lactate production, and extracellular acidification rate (ECAR), while increasing the oxygen consumption rate (OCR) in hypoxia-treated HCC cells. Additionally, GPC3 knockdown decreased the global lactylation and c-myc lactylation, which further decreased the protein stability and expressions of c-myc. This indicates that GPC3-mediated lactylation modification may be a new direction in HCC treatment in the future (33).

Meanwhile, in clear cell renal cell carcinoma (ccRCC), inactive von Hippel-Lindau (VHL) positively correlates with histone lactylation. Inactive VHL-triggered histone lactylation contributes to ccRCC progression by activating the transcription of platelet-derived growth factor receptor β (PDGFRβ), and PDGFRβ signaling in turn stimulates histone lactylation, forming an oncogenic positive feedback loop (31).

### 4.2 Impact on immune escape

Tumor immune escape refers to the situation where tumor cells are not detected, recognized, and killed by the human immune system. It is usually protected by the tumor microenvironment, which is a complex network composed of tumor cells, immune cells, endothelial cells, stromal cells, fibroblasts, and various cytokines (34, 35). Through interactions with stromal cells and immune cells, or by secreting cytokines, tumor cells can evade recognition and attack, suppress the host’s immune response, thereby transforming the tumor microenvironment into one favorable for the tumor, ultimately leading to tumor cell immune escape (36). The excessive accumulation of lactate within the TME is no longer regarded as a mere metabolic byproduct, but rather as a pivotal metabolic signal and epigenetic regulator that drives tumor immune escape (37, 38). Elevated lactate levels profoundly reshape immune cell metabolism, signaling pathways, and chromatin landscapes, thereby suppressing antitumor immune effector functions while favoring the expansion of immunosuppressive cell populations.

#### 4.2.1 Macrophages

Tumor-associated macrophages (TAMs) are highly plastic immune cells that can adopt pro-inflammatory M1 or immunosuppressive M2 phenotypes in response to microenvironmental cues (39–42). While M1-like macrophages support antitumor immunity, M2-like TAMs dominate in most tumors and promote immune suppression, angiogenesis, and metastasis. Increasing evidence indicates that tumor-derived lactate acts as a key metabolic signal driving macrophage polarization toward an M2-like, tumor-promoting state (43, 44).

In breast cancer models, *in vitro* assays and mouse xenografts reveal that lactate activates ERK/STAT3 signaling and stabilizes HIF-1α, thereby mechanistically driving the expression of pro-angiogenic factors like VEGF and immunosuppressive markers such as ARG1 (45). Lactate can also be sensed by the G protein–coupled receptor (GPR132), further reinforcing M2 polarization and facilitating tumor metastasis (46).

Lactylation has emerged as a pivotal mechanism linking lactate accumulation to macrophage functional reprogramming. Enhanced glycolysis in activated macrophages leads to increased histone lactylation, particularly H3K18la, which transcriptionally promotes M2-associated gene expression (47, 48). While clinical data show that histone lactylation correlates with pro-tumorigenic activity (29), causal evidence primarily stems from *in vitro* p300-mediated lactylation models. Similarly, the lactylation of metabolic enzymes, such as pyruvate kinase M2 (PKM2), has been shown to functionally facilitate the M1-to-M2 transition by modulating glycolytic flux (49).

#### 4.2.2 T cell

T cells are key lymphocytes that can directly eliminate target cells, regulate antibody production by B cells, produce cytokines, and respond to specific antigens and signals. Elevated lactate concentrations within the TME suppress T cell–mediated immune responses, impairing the cytotoxicity, chemotactic migration (50), and proliferation of both CD4^+^ and CD8^+^ T lymphocytes, and leading to reduced cytolytic activity and cytokine production (51).

Previous studies have demonstrated that high-lactate conditions markedly reduce T cell cytolytic activity and cytokine production, including interferon-γ (IFN-γ), tumor necrosis factor-α (TNF-α), and interleukin-2 (IL-2). *In vitro* assays show that the suppression is mediated in part by inhibition of p38 and JNK/c-Jun phosphorylation, thereby compromising cytotoxic T lymphocyte function (52). In parallel, lactate disrupts T cell metabolic homeostasis by depleting nicotinamide adenine dinucleotide (NAD^+^), which ultimately promotes T cell apoptosis. In ovarian cancer and other malignancies, *in vivo* evidence suggests that tumor-derived lactate further suppresses FIP200 expression and autophagy, exacerbating naïve T cell apoptosis and impairing antitumor immunity (53). Importantly, the regulatory effects of lactate on T cells are not uniformly inhibitory but are highly context-dependent, varying with lactate concentration, chemical form (lactic acid versus lactate anion), transporter usage, and the metabolic state of T cells. While lactic acid (low pH) is generally immunosuppressive, recent *in vitro* studies have proposed a noncanonical activation pathway where lactate anions may participate in TCR/CD3 signaling (54). In contrast, chronic exposure to high lactate levels, particularly through monocarboxylate transporter 11 (MCT11)–mediated uptake, imposes sustained metabolic stress that drives T cell dysfunction and exhaustion, thereby limiting antitumor immunity (55).

Lactate also reshapes CD4^+^ T cell differentiation through metabolic–epigenetic coupling mechanisms, skewing the Th17/Treg balance toward an immunosuppressive phenotype (56). CD8^+^ T cells are the principal effector cells in antitumor immunity, responsible for recognizing and eliminating malignant cells. Recent studies have demonstrated that lactic acid suppresses TCR/CD28-driven activation and reprograms cellular metabolism, resulting in reduced cytotoxic activity and cytokine secretion (57). In addition, lactate alters the balance of pyruvate entry into the TCA cycle, reshaping CD8^+^ T cell metabolism and suppressing their antitumor immune responses (58). Collectively, lactate orchestrates metabolic and signaling reprogramming to systemically dampen T cell–mediated antitumor immunity.

#### 4.2.3 Regulatory T cells

Regulatory T (Treg) cells play a pivotal role in maintaining an immunosuppressive tumor microenvironment, and a lactate-enriched metabolic milieu not only supports their expansion but also sustains their immunosuppressive function. Clinical correlations frequently link high lactate levels with increased Treg infiltration; however, mechanistic evidence from murine models has demonstrated that lactate acts as a functional substrate that drives Treg stability. Reducing intratumoral lactate levels has been functionally validated to limit Treg induction and restore antitumor immune responses (59). Tumor-infiltrating Treg cells can uptake lactate to upregulate FOXP3 and monocarboxylate transporter 1 (MCT1), thereby maintaining their immunosuppressive phenotype. In addition, lactate induces lactylation of MOESIN at Lys72, which enhances its interaction with transforming growth factor-β (TGF-β) receptor I and downstream SMAD3 signaling, leading to increased Treg cell stability and reinforced immunosuppressive activity (60, 61). Furthermore, *in vivo* assays reveal that by modulating CD4^+^ T cell differentiation, lactate increases the proportion of Treg cells while reducing the Th17/Treg ratio, thereby promoting the establishment of an immunosuppressive tumor microenvironment (62).

#### 4.2.4 Dendritic cell

Dendritic cells (DCs) play a pivotal role in initiating antitumor adaptive immunity through antigen uptake, processing, and presentation. However, the accumulation of lactate in the TME suppresses DC function through multiple mechanisms, thereby compromising immune surveillance and antitumor immune responses.

Lactic acid suppresses early DC differentiation and promotes a tumor-associated DC phenotype characterized by reduced antigen-presenting capacity, decreased IL-12 production, and increased IL-10 secretion. *In vitro* assays have demonstrated that lactate downregulates MHC-II and costimulatory molecules on DCs, impairing their ability to activate CD8^+^ T cells. *In vivo* evidence further indicates that inhibiting lactate production restores DC immunostimulatory activity and enhances antitumor immunity (63). The *in vivo* model further confirms that inhibiting lactic acid production can restore the immune stimulating ability of DCs and enhance the anti-tumor immune response. Tumor-derived lactate also engages GPR81 and is taken up via monocarboxylate transporters (MCTs) to suppress glycolysis and calcium signaling, inducing metabolic reprogramming that disrupts DC energy homeostasis, migration, and antigen processing capacity (64). Moreover, lactate activates the SREBP2 signaling axis, driving conventional DCs toward a mature regulatory DC (mregDC) state that preferentially traffics to tumor-draining lymph nodes and suppresses antigen cross-presentation and T cell activation (65).

#### 4.2.5 Other cells within TME

Beyond canonical immune populations, multiple non-immune and immune-supportive cell types within the tumor microenvironment critically contribute to lactate-driven immune escape. Tumor-derived lactate promotes the expansion and suppressive function of myeloid-derived suppressor cells (MDSCs) through metabolic reprogramming (66), inducing upregulation of ARG1, iNOS, and reactive oxygen species (ROS) (67), thereby establishing a durable immunosuppressive barrier in tumor cores (68, 69). In parallel, cancer-associated fibroblasts (CAFs) uptake and metabolize lactate to sustain a high-lactate niche and secrete CXCL12 (70), transforming growth factor-β (TGF-β), and extracellular matrix components, generating an immune-excluded stromal architecture that restricts dendritic cell and effector T-cell infiltration (71, 72). Lactate further acts on tumor-associated endothelial cells to drive aberrant angiogenesis and downregulate adhesion molecule expression, impairing immune cell transmigration and reinforcing metabolic–vascular immune exclusion (73). In addition, elevated lactate levels and acidic conditions suppress the metabolic fitness and cytotoxic capacity of natural killer (NK) cells, weakening innate immune surveillance (74). Collectively, lactate orchestrates a multicellular metabolic and spatial remodeling of the TME, establishing an environment for tumor immune evasion.

While these causal mechanisms are robustly supported by laboratory data, further clinical validation is required to determine the relative dominance of these pathways in the highly heterogeneous human TME.

### 4.3 Influence on tumor drug resistance

Drug resistance is a major challenge in cancer treatment, and lactylation may be involved in this phenomenon (75–77).

In hepatocellular carcinoma, upregulation of SPP1 promotes malignant progression and drug resistance by enhancing fatty acid metabolism, while inhibition of lipid oxidation or SPP1 knockdown attenuates these effects (78).

Bevacizumab has been proved effective in treating colorectal cancer (CRC), but higher level of lactylation confers drug resistance to bevacizumab which was associated with poor survival in CRC patients. Lactylation levels and H3K18la levels positively regulate the expression level of oncogene RUBCNL, thereby conferring CRC resistance to bevacizumab. When receiving bevacizumab, the glycolysis of hypoxic cancer cells may be further enhanced, followed by higher levels of histone lactylation and RUBCNL is transcriptionally upregulated by histone lactylation, which contributes to colorectal cancer cells survival and therapy resistance (79).

In addition, lactylation of non-histone proteins has emerged as a critical mechanism driving chemoresistance. According to the researches conducted by Cheng et al, their findings indicate that MRE11 lactylation promotes HR, and targeting MRE11 lactylation might be an effective strategy to overcome chemoresistance in cancers. Their assays are treated with NALA and LDHi respectively: NALA treatment increased protein lactylation and led cells to become resistant to cisplatin or olaparib treatment. By contrast, LDHi treatment decreased protein lactylation and sensitized cancer cells tocisplatin, olaparib, or etoposide. In addition, depletion of LDHA/B, which resulted in a significant reduction of lactate in cells, also markedly sensitized cancer cells to ola-parib. Thus, combining CBPi or LDHi with chemotherapy might be a potentially effective strategy to treat cancer with high levels of MRE11 K673 lactylation (80).

### 4.4 Effect on tumor invasion and migration

Accumulating evidence indicates that lactylation contributes to tumor invasion and migration by regulating autophagy, non-coding RNA networks, and cytoskeletal dynamics.

In prostate cancer, lactylation-associated autophagy-related genes such as baculoviral inhibitor of apoptosis repeat containing 5 (BIRC5) and neuregulin 2 (NRG2) have been implicated in malignant progression, suggesting that lactylation-mediated modulation of autophagy may influence tumor cell motility (81). In bladder cancer, lactylation-related regulatory networks involving the TRPM2-AS promote cell proliferation, migration, invasion, and epithelial–mesenchymal transition through the miR-195-5p/COP1 axis and activation of the PI3K/AKT signaling pathway (82). In pancreatic ductal adenocarcinoma, inhibition of glycolysis or suppression of LDHA reduces histone lactylation levels and significantly impairs tumor cell migration, whereas exogenous lactate partially rescues these effects (83).

## 5 Targeted lactate and lactylation for the treatment of tumors

With the in-depth study of TME, the crucial role of lactate and its metabolic products in tumor initiation and progression has gradually been revealed. Targeting lactate for cancer treatment is becoming a feasible strategy aimed at inhibiting the accumulation of lactate in tumor cells or disrupting its production mechanisms, thereby suppressing the metabolic adaptation of tumors. In recent years, several drugs targeting lactate and its related metabolic pathways, such as MCT1/MCT4 inhibitors and LDH inhibitors, have entered the preclinical research stage, offering new directions for cancer treatment (84). Lactylation, as an emerging post-translational modification, has become a potential target in cancer therapy. By targeting lactylation and its associated enzymes, it is possible to effectively regulate tumor cell metabolism and the immune environment, providing new therapeutic strategies and opening up possibilities for lactate and lactylation-targeted cancer therapies.

### 5.1 Targeting the production and transport of lactate

Accumulating evidence indicates that aberrant lactate metabolism is not merely a metabolic hallmark of cancer cells but a critical driver of tumor immune suppression and therapeutic resistance. Tumor cells exhibit an excessive dependence on glycolysis and lactate production, which supports their survival under hypoxic conditions and simultaneously reshapes the TME into an immunosuppressive and treatment-refractory niche. Consequently, restoring metabolic balance by targeting lactate production and utilization has emerged as a promising strategy to disrupt both immune evasion and drug resistance.

Lactate production and clearance are crucial components of tumor metabolism, involving the regulation of several key enzymes. LDH is one of the most critical enzymes, and its activity is usually significantly enhanced in tumor cells. By inhibiting LDH activity, lactate production can be effectively reduced, altering the metabolic state of tumor cells and inhibiting their proliferation. For example, certain small molecule inhibitors have shown the ability to specifically inhibit LDHA activity, directly disrupting redox homeostasis, and sensitizing tumor cells to cytotoxic stress, ultimately limiting tumor growth and metastatic potential (85, 86). LDHA inhibitors have demonstrated promising anti-tumor effects in preclinical models. However, their clinical translation remains limited. In terms of specificity, LDHA is also expressed in normal tissues, raising concerns about systemic toxicity. Moreover, tumor metabolic plasticity allows cancer cells to compensate for LDH inhibition by utilizing alternative substrates such as glutamine, which represents a major therapeutic challenge.

In addition to directly inhibiting lactate production, inhibiting lactate transport is also an effective approach to overcoming lactate-driven immune suppression and resistance. After lactate is produced, tumor cells need to export it through lactate transport proteins (such as MCT1 and MCT4) to maintain intracellular acid-base balance and prevent lactate accumulation within the cells. Inhibition of MCTs, particularly MCT1 inhibitors such as AZD3965, has shown anti-tumor activity by inducing intracellular lactate accumulation, metabolic stress, and acidification, ultimately suppressing tumor cell proliferation and enhancing sensitivity to therapy (87). MCT4 is especially important as it is responsible for transporting lactate from tumor cells to TME, preventing lactate build-up inside tumor cells which could lead to acidosis and impact on cell survival (88). Inhibition of MCT4 leads to intracellular lactate accumulation, metabolic imbalance, and intracellular acidification, conditions that are unfavorable for tumor cell proliferation and may promote tumor cell death by altering intracellular signaling pathways (89), and are associated with reduced resistance to therapy. From a translational perspective, MCT inhibitors have entered clinical trials, highlighting their feasibility. However, their specificity remains a concern due to the expression of MCTs in normal tissues. Additionally, compensatory upregulation of alternative transporters and metabolic pathways may limit long-term efficacy, representing key challenges in clinical application.

Targeting lactate production and transport not only disrupts tumor metabolism but also reshapes the tumor immune microenvironment. Lactate accumulation in the TME suppresses cytotoxic T cell and NK cell function while promoting regulatory T cell expansion and M2 macrophage polarization. However, since immune cells also rely on metabolic pathways involving lactate, achieving selective targeting without impairing normal immune function remains a critical challenge.

### 5.2 Targeting the regulation of lactylation

Accumulating evidence demonstrates that lactate dynamically drives lysine lactylation of histone and non-histone proteins in a glycolysis-dependent manner, thereby reshaping gene expression, protein function, and cellular adaptation. Histone lactylation regulates chromatin accessibility and transcriptional programs associated with tumor progression and immune modulation, whereas non-histone lactylation modulates protein stability, enzymatic activity, and signaling pathways, collectively facilitating tumor survival under metabolic and therapeutic stress.

Targeting lactylation is also a promising approach in cancer therapy. In ocular melanoma, correction of aberrant histone lactylation showed therapeutic efficacy both *in vitro* and *in vivo.* Histone lactylation contributed to tumorigenesis by facilitating YTHDF2 expression, which recognized and degraded m6A-modified PER1 and TP53 mRNAs. Inhibiting this process could potentially be a novel therapeutic strategy for ocular melanoma (30).

In gastric cancer, Sun et al. identified METTL16 as a key regulator of cuproptosis. Lactylation of METTL16 at K229 enhances FDX1 expression through m6A modification of FDX1 mRNA, thereby triggering cuproptosis under copper stress. Given the elevated copper and lactate levels in gastric tumors, targeting METTL16 lactylation in combination with copper ionophores and SIRT2 inhibitors may represent a promising therapeutic strategy (90).

In hepatocellular carcinoma, demethylzeylasteral (DML) suppressed the tumorigenicity of liver cancer stem cells by interfering with lactylation of a metabolic stress-related histone. DML inhibited H3 histone lactylation, specifically at H3K9la and H3K56la sites, which promoted tumorigenesis. In a nude mouse tumor xenograft model, the anti-liver cancer effects of DML were mediated by regulating H3 lactylation, supporting the feasibility of targeting lactylation in HCC (91).

Besides, Xie et al. observed a global Kla abundance profile in colorectal cancer (CRC) that negatively correlates with prognosis. Among lactylated proteins detected in CRC, lactylation of eEF1A2K408 resulted in boosted translation elongation and enhanced protein synthesis which contributed to tumorigenesis. By screening eEF1A2 interacting proteins, we identified that KAT8, a lysineacetyltransferase that acted as a pan-Kla writer, was responsible for installing Kla on many protein substrates involving in diverse biological processes. Deletion of KAT8 inhibited CRC tumor growth, especially in a high-lactic tumor microenvironment. Therefore, the KAT8-eEF1A2 Kla axis is utilized to meet increased translational requirements for oncogenic adaptation. As a lactyltransferase, KAT8 may represent a potential therapeutic target for CRC (92).

Collectively, targeting lactylation represents a novel and mechanistically grounded therapeutic strategy. However, several challenges remain, including the incomplete characterization of lactylation regulatory machinery, lack of highly specific inhibitors, and the context-dependent roles of lactylation in different tumor types. Addressing these issues will be essential for translating lactylation-targeted therapies into clinical practice.

### 5.3 Targeting lactate-related signaling pathways

Beyond its metabolic and epigenetic roles, lactate functions as an active signaling molecule that orchestrates tumor immune suppression and drug resistance through receptor-mediated pathways and lactylation-dependent modulation of intracellular signaling networks. Interference with the lactate-related signaling pathways occurs mainly through two mechanisms: inhibition of lactate receptors and interference with lactylation modification.

Firstly, lactate can bind to lactate receptors (such as GPR81) on the cell membrane, activating a series of signaling pathways that promote tumor cell growth and immune evasion. GPR81 is a major lactate receptor, and lactate concentrations are often elevated in TME. When GPR81 is activated, it triggers downstream signaling pathways such as cAMP/PKA, which increase tumor cell proliferation, migration and immunosuppressive effects, thereby promoting tumor malignancy (93). Therefore, targeting lactate receptors, especially using antagonists of GPR81, can effectively disrupt lactate-mediated signaling, reduce tumor cells’ ability to escape the immune system, and inhibit tumor proliferation and drug resistance (94). This strategy opens up new avenues for cancer treatment with significant potential, particularly in combination with immunotherapy and targeted therapies.

Secondly, lactate also regulates multiple key cellular signaling pathways (such as Wnt, Notch, HIF-1α, etc.) through lactylation modification of proteins, promoting the survival and maintenance of the malignant phenotype of tumor stem cells. This process is one of the reasons for tumor recurrence and metastasis (95). Under hypoxic and high-lactate conditions, lactylation enhances HIF-1α–driven transcriptional programs, reinforcing glycolytic metabolism and promoting survival under therapeutic stress. This metabolic–signaling feedback loop contributes to resistance to chemotherapy and radiotherapy by enabling tumor cells to tolerate oxidative stress and DNA damage.

In summary, the lactate-driven signaling pathway links metabolic reprogramming, immunosuppression, and treatment resistance closely together. Developing lactoyl inhibitors to interfere with lactate-mediated protein modifications can inhibit the proliferation and self-renewal of tumor stem cells, restore anti-tumor immune responses, and thereby reduce tumor recurrence and metastasis. However, the lack of highly selective inhibitors, incomplete understanding of lactylation-mediated signaling crosstalk, and tumor heterogeneity further complicate clinical translation. From a translational perspective, future efforts should focus on developing specific inhibitors, identifying predictive biomarkers, and optimizing combination strategies with immunotherapy and targeted therapies.

### 5.4 Remodeling acidic TME to restore antitumor immunity

The metabolic process of tumor cells often leads to the accumulation of a large amount of lactic acid in the tumor microenvironment, forming an acidic TME, which directly impairs the metabolic capacity, effector function and tumor infiltration ability of immune cells (96). Modifying TME, primarily by alleviating tumor acidosis and enhancing the tumor immune response, has become a potential therapeutic strategy (97).

By using alkaline agents (such as sodium bicarbonate) or targeting acidic environment-regulating factors like carbonic anhydrases (e.g. CAIX), lactate accumulation can be reduced, thereby restoring the normal pH level of TME. Drugs like sodium bicarbonate can neutralize acidic substances, inhibiting tumor cells from producing excess lactate, thus reducing the supportive role of the acidic environment on tumor cells (98). Inhibition of carbonic anhydrases (such as CAIX) can also effectively interfere with the acidification process of tumor cells, improving TME and providing new directions for treatment (99).

Lactate accumulation also helps tumors evade immune surveillance by inhibiting T cell activity. Lactate accumulation in TME negatively affects immune cells, especially T cells, reducing the effectiveness of immune responses (58). Therapeutic strategies targeting lactate accumulation aim to enhance immune cell function by improving the immune microenvironment and restoring tumor immunogenicity (100). For example, combining immune checkpoint inhibitors (such as PD-1/PD-L1 inhibitors) with lactate interventions can effectively enhance the anti-tumor ability of T cells, helping the immune system better recognize and eliminate tumor cells.

Collectively, remodeling the acidic tumor microenvironment represents a promising therapeutic strategy to restore anti-tumor immunity. Approaches such as buffering agents and inhibitors of acid-regulating enzymes have demonstrated encouraging results in preclinical studies and are supported by a clear mechanistic rationale. However, specificity remains a major concern, as systemic modulation of pH or acid-base balance may affect normal tissues and physiological processes. In addition, tumor heterogeneity and adaptive metabolic responses may limit therapeutic efficacy.

### 5.5 Combined treatment strategies

An important application of lactate-targeted therapy in tumors is its combination with traditional treatment methods such as chemotherapy, radiation therapy and targeted therapies to enhance efficacy and overcome tumor resistance. Excessive lactate accumulation in tumors supports cancer stem cell survival and promotes resistance to chemotherapy and radiation. By limiting lactate accumulation and restoring pH balance in the TME, lactate-targeted interventions can sensitize tumor cells to cytotoxic agents and improve radiotherapy efficacy by alleviating hypoxia and enhancing oxidative stress–induced DNA damage (101).

Lactate-targeted therapies can also be combined with immunotherapy, particularly immune checkpoint inhibitors (such as anti-PD-1/PD-L1 antibodies) or CAR-T cell therapy, to further enhance the immune system’s ability to attack tumors (102–108). Elevated lactate levels in the TME suppress immune cell function, particularly that of T cells, thereby limiting the effectiveness of immune checkpoint inhibitors and adoptive cell therapies. Targeting lactate metabolism can reverse this immunosuppressive state, enhance T-cell activity, and improve responses to immune checkpoint blockade (109). Moreover, remodeling the lactate-rich microenvironment creates more favorable metabolic conditions for CAR-T cells, thereby enhancing their antitumor efficacy (110).

Overall, by combining with chemotherapy, radiation therapy, and targeted therapy, lactate-targeted treatment can overcome tumor resistance and improve treatment sensitivity. When combined with immunotherapy, it can restore immune responses and enhance the anti-tumor functions of immune cells, providing a more comprehensive and effective treatment plan (111). These innovative treatment strategies offer new hope for comprehensive cancer treatment.

### 5.6 Precision and personalized treatment

Personalized cancer treatment refers to treatment plans tailored to each patient based on the genetic characteristics, molecular features and pathological conditions of their tumor (112, 113). The key lies in precisely targeting specific mutations in the tumor, avoiding the blind approach of traditional treatments, maximizing therapeutic efficacy and minimizing side effects. Personalized treatment can improve the accuracy of treatment, enhance patients’ survival rates and quality of life, and help overcome issues such as tumor resistance and recurrence (114, 115).

Tumor metabolic states are often diverse, with varying levels of lactate accumulation depending on the tumor type and its microenvironment. Given the heterogeneity of tumor metabolism, personalized lactate-targeted therapy becomes particularly important. By precisely assessing the metabolic characteristics of a tumor, more effective treatment plans can be tailored for different patients (116). Lactate-targeted interventions can be carried out through various approaches, such as inhibiting lactate production, promoting lactate clearance, or altering the acidity of TME to reduce the negative effects of lactate accumulation.

In practical clinical applications, technologies such as advanced metabolomics and molecular imaging allow physicians to better understand the metabolic characteristics of tumors and thereby develop personalized lactate-targeted treatment strategies for patients (117). For example, for tumors with high lactate production, drugs that inhibit lactate generation can be chosen, while for tumors that rely on lactate accumulation to promote growth, other approaches can be used to regulate their metabolic pathways, controlling lactate accumulation and reducing the malignancy of the tumor.

In summary, personalized lactate-targeted therapy not only helps to improve treatment efficacy and reduce unnecessary side effects, but also may open up new directions in cancer treatment. Especially in the context of precision treatment targeting tumor metabolic characteristics, intervention in lactate metabolism may become a key breakthrough in cancer therapy.

### 5.7 Challenges and future perspectives

Despite significant advances in understanding lactate metabolism and lactylation in cancer, several key challenges still limit their clinical translation. First, lactate-related regulatory networks span multiple layers of tumor biology, including metabolism, epigenetics, signaling, and immune modulation, and exhibit strong context dependency and tumor heterogeneity, complicating target selection and therapeutic prediction. Second, the lack of specificity remains a major concern, as many targets involved in lactate metabolism and lactylation, such as LDHA, monocarboxylate transporters (MCTs), and acetyltransferases, also play essential roles in normal physiological processes, raising the risk of systemic toxicity. Third, the regulatory mechanisms of lactylation are still incompletely understood, particularly regarding the identification of specific “writers,” “erasers,” and “readers,” as well as their site-specific and context-dependent functions. Finally, tumor metabolic plasticity enables cancer cells to bypass lactate-targeted interventions by activating alternative metabolic pathways, thereby limiting long-term therapeutic efficacy.

Future research should focus on improving the feasibility and specificity of lactate-targeted therapies while enhancing their translational potential. The development of highly selective inhibitors and tumor-specific targeting strategies will be essential to minimize off-target effects. Advances in multi-omics technologies and systems biology may facilitate the identification of predictive biomarkers, enabling patient stratification and precision medicine approaches. In addition, combination strategies integrating lactate-targeting interventions with immunotherapy, metabolic modulation, or epigenetic therapies are likely to enhance therapeutic efficacy by simultaneously targeting multiple hallmarks of cancer. A deeper understanding of lactylation dynamics and its interplay with other regulatory mechanisms will further accelerate the development of effective and clinically applicable therapeutic strategies.

## 6 Conclusion

Metabolism plays a crucial role in the occurrence and development of tumors, especially in tumor cell energy metabolism, acid-base balance and immune evasion mechanisms. A large body of research has revealed the close connection between lactate metabolism and other metabolic pathways in tumor cells (118–120). For example, lactate is not only a by-product of glycolysis, but is also involved in fatty acid metabolism, amino acid metabolism and others. Lactate participates in regulating the metabolic reprogramming of tumor cells through its interaction with intracellular metabolic networks. Moreover, lactate interacts with factors such as hypoxia and inflammation in TME, jointly driving the malignant transformation of tumors (121).

As a new post-translational modification, lactylation has gradually gained attention in the fields of metabolism and tumor research. Lactylation refers to the process by which lactate molecules bind to lysine residues of proteins to form lactylation modifications, which is similar to other types of post-translational modifications such as acetylation and phosphorylation (122). Lactylation not only participates in the regulation of tumor cell energy metabolism, but also plays an important role in tumorigenesis, development, metastasis and immune evasion mechanisms (123).

Lactylation also plays a key role in tumor immune evasion. Tumor cells secrete lactate, acidifying TME, thereby reducing the activity and function of immune cells and helping the tumor escape surveillance by the host immune system (124). Lactylation modification may further promote this process by regulating the function of immune-related proteins. For example, lactylation may modify immune checkpoint molecules or tumor-associated macrophages, enhancing their immunosuppressive function (125).

Lactate metabolism targeted therapies have become one of the forefront areas of tumor treatment research. In preclinical studies, therapeutic agents targeting lactate metabolism have made some progress. For example, inhibitors targeting key enzymes in lactate metabolism, such as LDH and MCTs, have shown potential in inhibiting tumor growth in *in vitro* experiments and animal models. The results of preclinical studies have laid the foundation for the development of therapies targeting lactate metabolism. In the future, drugs that inhibit lactate production or transport may be developed to reduce tumor cell metabolism. These drugs may be used alone or in combination with other therapeutic strategies such as chemotherapy and immunotherapy to enhance therapeutic effects (126).

In research on lactate metabolism-targeted therapy, lactylation as a new regulatory level may become a potential therapeutic target. By intervening in the lactylation modification process, it may effectively suppress tumor cell metabolic reprogramming, inhibit tumor growth and metastasis, and enhance the effectiveness of immunotherapy (127). Additionally, lactylation modifications may cooperate with other post-translational modifications such as acetylation and phosphorylation, providing new insights for the development of more precise tumor treatment strategies.

However, therapy targeting lactate metabolism also faces a number of challenges in clinical applications. Firstly, lactate is an important product of normal cellular metabolism, and excessive inhibition of lactate production or transport may adversely affect normal tissues, especially high-energy-demand tissues such as heart and muscle, potentially leading to side effects. In addition, the regulation of lactate metabolism may trigger compensatory responses in other metabolic pathways, leading tumor cells to restore energy metabolism through other mechanisms. Therefore, how to precisely target tumor-specific lactate pathways while minimizing the impact on normal cells is a significant challenge for lactate metabolism-targeted therapy.

With a deeper understanding of the mechanisms of lactate metabolism, researchers will be able to develop more precise targeted drugs to intervene in specific lactate metabolic pathways, thereby inhibiting tumor growth and metastasis. In addition, personalized treatment strategies for lactate metabolism are expected to be applied clinically, combining the molecular characteristics and metabolic status of tumors to provide tailored treatment plans for patients. However, several technical and biological challenges, such as drug selectivity and side effect control, still need to be overcome before lactate metabolism-targeted therapy can be widely applied in the clinic. As research continues to advance, the prospects of lactate metabolism regulation in tumor treatment are vast, potentially opening a new avenue for cancer therapy.
